# Biosensor Technology: Advances and Applications in Livestock Infectious Disease Diagnosis

**DOI:** 10.3390/vetsci12010023

**Published:** 2025-01-07

**Authors:** Yanan Zhao, Lu Zhang, Aihua Wang, Dong Zhou

**Affiliations:** 1 College of Veterinary Medicine, Northwest A&F University, Yangling 712100, China; zyn991221@163.com; 2 Key Laboratory of Animal Biotechnology, Ministry of Agriculture and Rural Affairs, Northwest A&F University, Yangling 712100, China; 3 Department of Animal Engineering, Yangling Vocational & Technical College, Yangling 712100, China; luzhang2021@nwafu.edu.cn

**Keywords:** veterinary obstetrics, biosensors, reproductive diseases, diagnostic technologies, reproductive health

## Abstract

Biosensors have revolutionized the diagnosis of infectious diseases in livestock by offering sensitive and rapid detection methods for important pathogens. These devices, particularly luciferase-based biosensors, convert biological responses into quantifiable signals, enabling the real-time, non-invasive monitoring of critical biomarkers. This review explores the development and advantages of biosensors, focusing on their applications in detecting important livestock pathogens, including bacteria, parasites, and viruses. By comparing biosensors with traditional diagnostic methods, the review highlights their superior sensitivity and specificity in diagnosing obstetric infections. Furthermore, it discusses the broader utility of biosensors in monitoring gene expression, protein localization, and cellular events. Together, biosensors provide new tools for the timely diagnosis of infectious diseases in livestock and are critical to improving livestock health.

## Simple Summary

Biosensors address the critical problem of diagnosing infectious diseases in livestock, where the early and accurate detection of pathogens like *Brucella* and *Toxoplasma gondii* is essential for preventing significant reproductive health issues. This review aims to explore the development, advantages, and practical applications of biosensors, particularly those based on luciferase technology, which detect biological signals and translate them into measurable data for real-time, non-invasive monitoring. The review finds that biosensors offer superior sensitivity and specificity over traditional diagnostic methods, making them exceptionally effective for detecting obstetric infections. Furthermore, this study highlights the broader potential of biosensors in tracking gene expression, protein movements, and cellular processes, supporting diverse areas of veterinary and medical research. These advancements in biosensor technology enable faster, more accurate diagnosis and timely management of reproductive diseases, ultimately benefiting society by improving animal health, supporting the agricultural sector, and safeguarding food resources.

## 1. Introduction

**Figure 1:**
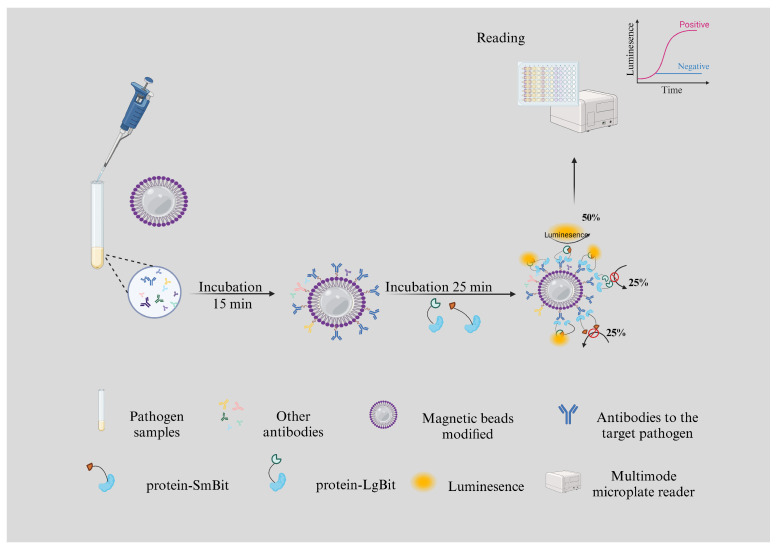
A schematic diagram of the lysis– NanoLuc complementation assay for the detection of the antibody of interest. Protein A/G magnetic beads are enriched for antibodies in serum. Protein A/G magnetic bead–serum complexes were incubated with LgBiT/SmBiT protein fusion probes. One Fab arm of the IgG antibody binds to the LgBiT sensor, and the other Fab arm binds to the SmBiT sensor, recombines with the NanoBiT luciferase, and generates an active luciferase signal. Antibodies can also specifically bind to LgBiT or SmBiT fusion sensors without luciferase activity, resulting in a 50% functional enzyme reconstitution rate. The luminescence signal is detected through a multimode microplate reader to determine the negative and positive values of the sample.

Biosensors have emerged as essential tools in the field of molecular biology and bioanalytics, offering highly sensitive and specific methods for detecting and quantifying a wide range of biomolecules. These devices are valued for their ability to convert a biological response into a detectable signal, directly correlating with the concentration of the target molecule [1,2,3]. Since the discovery and initial application of luciferase in the 1960s, these biosensors have evolved significantly, becoming indispensable in diverse areas such as gene expression monitoring, protein localization, and the diagnosis of various pathogens [4,5,6]. The capacity of electrochemical biosensors and luciferase-based biosensors to provide non-invasive, real-time, and quantitative data has positioned them at the forefront of biological research and diagnostic technologies [2,7]. Biosensors have played an increasingly important role in diagnosing key pathogens of livestock [8]. For instance, biosensors have been effectively used in the detection of *Brucella*, *Toxoplasma gondii*, and *African Swine Fever (ASF)* infections [9,10,11,12]. The application of biosensors in their detection has enhanced the ability to identify infections quickly and accurately [8,13,14]. A recently developed biosensor used for detecting African Swine Fever virus is NanoBiT. This biosensor employs a split-luciferase complementation system based on NanoLuc binary technology. NanoBiT consists of a large fragment (LgBiT) and a complementary 11-amino-acid peptide (SmBiT). These subunits have a low affinity for each other but form a functional luciferase when brought close together, producing a strong luminescent signal in the presence of furimazine [11,12]. Using antigen–antibody interactions, this system allows for the rapid, sensitive, and quantitative detection of antigens or their specific antibodies in clinical samples, as shown in Figure 1.

This review aims to provide a comprehensive overview of biosensor technologies in the diagnosis of infectious diseases in animals, highlighting their development, advantages, and specific applications. The key objectives of this review are to (1) explore the molecular mechanisms underlying the operation of various biosensors, (2) compare these biosensors with traditional diagnostic technologies in terms of performance and applicability, and (3) discuss pathogen detection in livestock, such as bacteria, parasites, and viruses [15,16,17]. By analyzing recent advancements and case studies, this review seeks to provide valuable insights into the full potential of biosensor technologies for diagnosing animal diseases.

## 2. Molecular Mechanisms of Biosensors

Biosensors operate by leveraging biorecognition elements to detect target molecules, converting biological reactions into measurable signals through a transducer and amplifier. For example, luciferase-based biosensors emit light in proportion to the target molecule concentration, enabling highly sensitive detection [18,19]. Initially used for simple luminescence detection, luciferase biosensors have advanced into tools for real-time, non-invasive monitoring across various applications, including gene expression and pathogen detection [18,20,21]. Recent developments in fusion–fission biosensors have improved their dynamic range and specificity, enhancing their precision in monitoring molecular events [22,23].

### 2.1. Basic Principles of Biosensor Operation and Mechanisms of Action of Different Biosensors

Biosensors function by combining biological reactions with physical signal transduction to detect specific target molecules, enabling real-time and sensitive quantitative analysis [24,25]. The key operating principle involves biorecognition elements (such as enzymes, antibodies, or nucleic acids) interacting specifically with target molecules, triggering a series of signal generation processes [26,27]. These reactions are converted into electrical, optical, or chemical signals for qualitative and quantitative analysis [28,29]. NanoBiT biosensors employ a split-luciferase complementation system based on NanoLuc binary technology. NanoBiT consists of a large fragment (LgBiT) and a complementary 11-amino-acid peptide (SmBiT). These two subunits have a low affinity for each other but form a functional luciferase when brought close together, producing a strong luminescent signal in the presence of furimazine [11,12]. The mechanisms behind using electrochemical biosensors for pathogen detection involve the use of electrochemical transducers combined with biorecognition elements to detect specific pathogens. The electrochemical transducer converts a biological response into an electrical signal, which can be measured and analyzed. Biorecognition elements, such as enzymes, antibodies, or bacteriophages, selectively bind to the target pathogens, causing a change in the electrical properties of the transducer. This change in the electrical signal is then correlated to the presence and concentration of the pathogen, enabling accurate and rapid detection [30]. Biorecognition elements determine the biosensor’s specificity and sensitivity [30]. Enzymes catalyze biochemical reactions, antibodies detect antigens, and nucleic acids enable gene detection [31]. Signal capture and amplification are critical steps, as they determine the sensor’s performance and detection limits [18].

### 2.2. Development of Luciferase-Based Biosensors: From Early Applications to Modern Technologies

Luciferase-based biosensors have demonstrated remarkable functionality and potential in various applications. The early applications of these biosensors primarily focused on their use in detecting biological processes through luminescence [32]. For instance, firefly luciferase was one of the first enzymes utilized in biosensing, enabling researchers to monitor gene expression and cellular activities by quantifying light emitted during the enzymatic reaction with luciferin [33,34]. This capability allowed for real-time observations of biological processes, significantly advancing our understanding of cellular mechanisms. However, these early applications were not without limitations. The sensitivity and specificity of the assays were often inadequate, leading to challenges in accurately measuring low-abundance targets [9]. Furthermore, the stability of luciferases in complex biological environments was a concern, as enzymatic activity could be affected by factors such as pH and temperature variations. Despite these challenges, the foundational work laid out by early luciferase biosensors paved the way for more sophisticated applications in modern diagnostics and research [35].

The evolution of luciferase technologies has led to significant advancements in sensitivity, specificity, and versatility. Modern luciferases, such as NanoLuc and Gaussia luciferase, have been engineered to exhibit improved kinetic properties and stability, allowing for more reliable and sensitive detection of biological events [36]. These advancements have enabled the development of novel biosensing applications, including the real-time monitoring of cellular processes and the high-throughput screening of drug candidates [37,38]. Additionally, modern luciferase systems often incorporate split-luciferase technology, which enhances the ability to study protein–protein interactions in living cells [39]. This innovative approach allows researchers to visualize and quantify interactions in real time, providing valuable insights into cellular signaling pathways and disease mechanisms. The continuous optimization of luciferase enzymes and their applications underscores the importance of these technologies in advancing biomedical research and diagnostics. In pathogen detection, luciferase biosensors detect pathogen-specific nucleic acids or proteins, enabling the rapid diagnosis of bacterial and viral infections [40]. For instance, luciferase-based sensors have been used for the rapid detection of pathogens like Brucella and Toxoplasma, significantly improving the speed and accuracy of diagnoses, thus contributing to better management of animal disease outbreaks [41,42]. In veterinary science, luciferase biosensors have gained popularity, especially in the areas of reproductive health and infectious disease control [43,44]. These sensors enable the real-time monitoring of pathogen infections in animals, providing crucial data for early diagnosis and intervention.

## 3. Comparison with Traditional Diagnostic Techniques

This section will focus on comparing biosensors with traditional diagnostic methods to evaluate the strengths and limitations of both approaches.

### 3.1. Traditional Diagnostic Tools Used in Veterinary Pathogen Infections

In the field of livestock pathogen detection, traditional diagnostic methods primarily rely on laboratory-based techniques, including culture methods, serological tests (such as the enzyme-linked immunosorbent assay, ELISA), and molecular detection techniques like the polymerase chain reaction (PCR). These methods are mainly used to detect reproductive diseases and related infections in livestock. For example, ELISA is widely applied to detect antibodies in livestock serum, identifying pathogens responsible for reproductive diseases, such as Brucella and Toxoplasma [45]. Additionally, PCR, known for its high sensitivity and specificity, is employed to detect pathogen nucleic acids, providing accurate results within a short timeframe [46]. PCR is particularly effective in identifying pathogens that cause infertility or miscarriage in livestock, such as Streptococcus [47,48]. Although the accuracy and reliability of these traditional diagnostic tools have been validated in numerous studies, they often require complex laboratory equipment and extended processing times. For instance, while bacterial cultures remain the gold standard for pathogen detection, it typically takes several days to yield results [49]. Similarly, ELISA testing often involves time-consuming sample preparation, and the results may be affected by variable antibody responses, leading to decreased sensitivity and specificity [50]. Consequently, the application of these traditional methods in veterinary practice generally depends on centralized laboratory environments, making it difficult to provide rapid, on-site diagnostics. Another limitation is the reduced sensitivity of these methods when detecting early-stage infections or pathogens present at low levels. While PCR is sensitive, samples with very low pathogen loads may require multiple amplification cycles or complex sample preparation [51]. Moreover, traditional serological tests like ELISA may struggle to detect pathogens during early infections, particularly when antibody concentrations are low and the immune response is not fully developed. Overall, while traditional diagnostic tools are theoretically accurate in controlled laboratory settings, their time requirements, equipment needs, and reliance on skilled technicians limit their broader use in veterinary clinics. As modern veterinary practice increasingly demands real-time, convenient diagnostics, the limitations of traditional methods have prompted a search for faster, portable, and more reliable alternatives, such as biosensors.

### 3.2. Performance Metrics: Sensitivity, Specificity, and Accuracy

The performance of diagnostic tools is typically evaluated using three key metrics: sensitivity, specificity, and accuracy. Sensitivity refers to the tool’s ability to correctly identify positive cases, i.e., the proportion of true positive results when the disease or pathogen is present. High sensitivity reduces the likelihood of false negatives, particularly in cases where pathogen concentrations are low or during the early stages of infection [52]. For instance, PCR exhibits remarkable sensitivity in the early detection of Brucella infections [53]. Specificity, on the other hand, measures the tool’s ability to correctly identify negative cases, meaning its capacity to yield negative results when the disease is absent. High specificity minimizes the risk of false positives, thereby reducing the occurrence of misdiagnoses. For example, although ELISA is widely used, its specificity can be affected by cross-reactivity with antigens, leading to a higher risk of false positives [54]. Accuracy represents the overall performance of a diagnostic tool, combining both sensitivity and specificity to reflect the correctness of the test results. Traditional methods like bacterial cultures are considered the “gold standard” due to their high accuracy, but they are time-consuming and reliant on laboratory infrastructure. In veterinary obstetrics, biosensors, by enabling real-time detection, show significant potential in improving accuracy while reducing the incidence of false positives and false negatives.

### 3.3. Advantages and Limitations of Biosensors Compared to Traditional Methods

Biosensors offer several distinct advantages over traditional diagnostic methods, particularly in terms of speed, real-time monitoring, and potential for on-site use without requiring sophisticated laboratory equipment. One of the most significant benefits of biosensors, especially luciferase-based systems, is their ability to provide non-invasive, highly sensitive detection, along with real-time feedback. This allows for rapid diagnosis in situations where time is critical, such as during the early stages of infection or when monitoring disease progression in livestock. Additionally, biosensors are highly versatile and can be adapted for various diagnostic purposes, including the detection of pathogens, metabolic markers, and even specific gene expression levels [55,56]. Despite these advantages, biosensors have several limitations. One major challenge is the need for optimization in complex biological environments, where factors such as background interference, pH, and temperature fluctuations can affect sensor performance [57,58]. Furthermore, long-term stability is another concern, as biosensors may lose sensitivity over extended periods, particularly in field settings [59]. Another limitation is the high cost associated with developing and producing highly specialized biosensors, which may restrict their widespread adoption in resource-limited or smaller veterinary practices [57,60]. Overall, while biosensors have demonstrated immense potential to outperform traditional methods in terms of speed and sensitivity, their limitations necessitate further research and development to ensure broader applicability and cost-effectiveness in practical settings, as shown in Table 1.

## 4. Applications of Biosensors in Livestock Infectious Pathogen Detection

### 4.1. Applications of Biosensors in the Detection of Viral Pathogens in Livestock

African Swine Fever (ASF), a devastating infectious disease threatening the global swine industry, exhibits a mortality rate of up to 100% upon infection. Although ASF itself does not directly cause obstetric diseases, its impact on the immune system, pregnancy management, and overall health may indirectly lead to reproductive issues. Recently, Zhang Z. et al. developed a NanoBiT biosensor-based immunoassay for ASFV IgG detection, using p30-fused LgBiT/SmBiT probes to reconstitute nanoluciferase upon antibody binding. This assay exhibited 16-fold higher sensitivity than ELISA, with 98.19% concordance to clinical samples and no cross-reactivity, offering a rapid, specific, and efficient method for ASFV antibody detection in clinical diagnostics [66]. Beyond ASFV, biosensors have been widely applied for detecting various viral pathogens in livestock, leveraging their rapid, sensitive, and highly specific diagnostic capabilities. Avian influenza virus (AIV) is a highly contagious pathogen with significant impacts on public health and animal health. Its highly pathogenic subtypes, such as H5 and H7, can cause severe disease and high mortality rates in poultry, with the added risk of cross-species transmission to humans, potentially leading to influenza pandemics [67]. The rapid detection and surveillance of AIV are critical for controlling the spread of outbreaks. A functionalized poly-crystalline silicon nanowire field-effect transistor (poly-SiNW FET) has been developed for the specific detection of target viral DNA. This biosensor employs complementary DNA capture probes immobilized on the nanowire surface to detect the hybridization of target DNA, such as the H5 subtype, through measurable electrical signal changes [68]. The sensor’s fabrication process is low-cost and compatible with existing semiconductor manufacturing techniques and eliminates the need for expensive tools like electron-beam lithography. It achieves high sensitivity in the femtomolar (fM) to picomolar (pM) range, with excellent specificity. These attributes, coupled with its portability, make the poly-SiNW FET a promising tool for on-site rapid diagnostics and pathogen surveillance.

Bovine viral diarrhea virus (BVDV) is the causative agent of bovine viral diarrhea, one of the most economically significant livestock diseases globally. The cornerstone of BVD control programs is the detection and elimination of persistently infected (PI) cattle, which serve as a continuous source of disease transmission [69,70]. A G-quadruplex-based aptasensor has been developed for the specific detection of BVDV-1 using a rationally designed G-quadruplex aptamer (Apt31) in combination with gold nanoparticles (AuNPs). The AuNPs are electrostatically linked with Apt31, and in the presence of BVDV, the formation of an aptamer–BVDV complex induces a salt-stimulated color change in the AuNPs from red to purple-blue, enabling rapid colorimetric detection [71]. The sensor exhibits high sensitivity with a detection limit as low as 0.27 copies/mL, comparable to the performance of qPCR methods. Additionally, the aptasensor demonstrates excellent specificity, as confirmed by cross-reactivity testing and plasma sample validation, achieving 90% precision and 94% accuracy. These advancements highlight the versatility and efficiency of biosensors in addressing diverse diagnostic challenges posed by viral pathogens in livestock, underscoring their potential for broader applications in both laboratory and field settings.

### 4.2. Applications of Biosensors in the Detection of Parasitic Pathogens in Livestock

*Toxoplasma gondii*, an intracellular parasite, primarily uses cats as its definitive host and is transmitted via cat feces, infecting other animals and subsequently spreading to humans through various routes [72]. Given its global prevalence, controlling its transmission is crucial. To address this issue, S. Anli et al. demonstrated the development of an antibody-based electrochemical biosensor for the direct detection of *T. gondii*, offering potential applications in disease monitoring and management [73,74]. The biosensor utilizes a screen-printed electrode modified with chitosan and gold nanoparticles, with anti-*T. gondii* antibodies immobilized on the electrode surface using glutaraldehyde as a cross-linker. Surface characteristics were analyzed through differential pulse voltammetry, cyclic voltammetry, and electrochemical impedance spectroscopy, while scanning electron microscopy was used to study the surface morphology of the immunosensor. The biosensor’s linear working range and detection limit were determined, and its performance was validated for detecting *T. gondii* in synthetic serum samples. This innovative approach holds promise for developing sensitive and specific diagnostic tools for *T. gondii* infections, which is critical for effective disease prevention. In addition to *Toxoplasma gondii*, biosensors have shown great potential in the detection of other parasitic pathogens, such as *Trichinella spiralis* and *Trypanosoma* spp. Trichinella spiralis, a zoonotic nematode transmitted through the consumption of undercooked meat, has been targeted using quantum dot-based fluorescent biosensors. These biosensors employ fluorescent quantum dots conjugated with specific antibodies to detect *T. spiralis* antigens with high sensitivity and specificity, providing a rapid and accurate diagnostic tool [75].

*Trypanosoma* spp., the causative agents of trypanosomiasis, pose significant threats to livestock and human health in endemic regions. Recent advancements include the development of a laboratory prototype biosensor utilizing fiber optic spectrometry [76]. This system measures the ultraviolet–visible–near–infrared (UV–Vis–NIR) absorbance of murine blood to detect infection-related changes in blood chemistry based on the Beer–Lambert Law. While the prototype showed a strong correlation between Plasmodium infection and spectral absorption at 650 nm, no significant spectral changes were observed for Trypanosoma infection in the current dataset. Nonetheless, this approach highlights the potential of spectrometric biosensors for non-invasive diagnostics. Future modifications and optimizations could enhance its sensitivity for detecting Trypanosoma infections, offering innovative tools for field and laboratory settings. Together, these advancements in biosensor technology provide effective and innovative methods for monitoring and controlling parasitic infections in livestock and human populations, addressing critical challenges in global public health.

### 4.3. Applications of Biosensors in the Detection of Bacterial Pathogens in Livestock

*Brucella* species are primarily transmitted to humans through direct contact with infected animals or the consumption of contaminated animal products, especially unpasteurized dairy products. Among the eight recognized *Brucella* species, six are known to infect humans, with *B*. *suis, B*. *melitensis*, and *B*. *abortus* exhibiting the highest pathogenicity [77,78]. The incubation period for brucellosis typically ranges from 7 days to several months, though it may be shorter with high-dose aerosol exposure. Although the untreated fatality rate is low (2–5%), the bacterium’s low infectious dose (10–100 organisms) and prolonged viability make it a significant pathogen [78,79]. Clinical manifestations vary depending on the *Brucella* species and individual patient factors, with a symptom onset that can be either gradual or sudden [80]. Traditional veterinary clinical diagnostic methods for Brucella detection, such as ELISA and PCR, are effective; however, they often require long detection times and reliance on laboratory equipment [64,65]. The use of nanoflake-based biosensors has shown significant advantages for the rapid detection of *Brucella*, particularly in the early stages of infection. Our research developed a NanoBiT-based luminescent assay using *Brucella Omp16* fusion probes for antibody detection. This system enhances sensitivity by 128-fold over indirect ELISA, achieving 98.55% concordance with commercial kits. Protein A/G beads capture serum antibodies, forming immune complexes that reconstitute NanoBiT luciferase and produce bioluminescence, offering a rapid, sensitive, and specific diagnostic tool for *Brucella* and other bacterial diseases.

*Escherichia coli* (*E. coli*), a Gram-negative bacterium, is widely found in the intestines of warm-blooded animals. While most strains are harmless or commensal, certain pathogenic strains, such as *E. coli O157*:*H7*, can cause severe diseases. In veterinary clinical settings, *E. coli*-induced mastitis remains a significant threat to dairy cattle, adversely affecting animal welfare and resulting in substantial economic losses [81]. A carbon nanotube field-effect transistor (FET)-based biosensor has been developed for the direct detection of trace amounts of bacterial genomic DNA. By immobilizing specific oligonucleotide probes on the FET array, this biosensor captures genomic DNA from *E. coli O157*:*H7*, resulting in significant threshold voltage (Vth) changes. In contrast, signals from non-target strains, such as *E. coli O45*, remain almost unchanged, demonstrating excellent sensitivity and specificity. This method requires minimal sample preparation and eliminates the need for PCR amplification or molecular labeling, providing an efficient option for genomic detection [82].

As another common bacterial pathogen in veterinary clinical practice, *Salmonella* poses significant challenges. Developing portable and user-friendly biosensor platforms capable of the label-free detection of diagnostic markers in undiluted animal sera offers great convenience for clinical applications. M. Ewald and colleagues designed a portable and easy-to-use biosensor platform utilizing a 1-lambda reflectometer for the label-free detection of anti-*Salmonella* antibodies [83]. This sensor immobilizes lipopolysaccharides (LPSs) from *Salmonella Typhimurium* onto the sensor’s sensitive layer to directly capture target antibodies through serological detection. The platform demonstrated excellent sensitivity and selectivity in undiluted sera and showed reproducibility both within and between chips, with recovery rates ranging from 99% to 117%. This biosensor provides a portable and efficient solution for the rapid detection of complex samples. These biosensor technologies demonstrate significant advantages in detecting *Brucella*, *Escherichia coli*, and *Salmonella*, covering multiple levels of detection, ranging from bacterial antibodies to genomic DNA. These approaches not only reduce detection time but also enhance sensitivity and specificity. With continued advancements in biosensor development, their applications in rapid clinical diagnostics and on-site detection are expected to expand further, offering broad prospects for future implementation.

## 5. Conclusions

Biosensors, as an emerging diagnostic tool, already haveapplication prospects in veterinary medicine and public health. While technical challenges remain, continuous innovation and optimization will ensure that biosensors become a key technology for advancing global animal health and public health in the future.

## Figures and Tables

**Table 1:** Comparison of common traditional detection methods and biosensors in pathogen detection.

Detection Method	Target	Laboratory or Point-of-Care (POC)	Quantitative	Test Charge	Advantage	Defect
RT-PCR	Nucleic acid	Laboratory	Semi-quantitative	High	1. Highly specific and sensitive 2. Suitable for early infection	1. Requires sample preparation and purification 2. Needs specific reagents and requires sophisticated and expensive machines 3. Needs skilled personnel and chances of false results are higher for mixed infection cases 4. Longer analysis time (~50 min to 4 h) and not suitable for large population 5. Not suitable for large-scale screening for multiple samples
ELISA	Antigen Antibody	Laboratory	Semi-quantitative	High	1. Suitable for monitoring the immune response 2. Suitable for sero-surveillance	1. Requires sample preparation and purification 2. Low specificity and high risk of cross-reactivity 3. Longer analysis time (~2 to 5 h) 4. Not suitable for large-scale screening for multiple samples
Electrochemical Biosensor	Any analyte depending on the biorecognition element	Laboratory or POC	Yes	Low	1. Rapid response time (~10 s to 1 h) 2. Highly specific 3. No need for complex reagents or sample preparation 4. Miniaturization capability	1. Sample matrixes affect the sensitivity of assay 2. Low stability
NanoBiT Biosensor	Antigen Antibody	Laboratory or POC	Semi-quantitative	Low	1. Rapid response time (30 min to 1 h) 2. Detects targets in the nanomolar (nM) to picomolar (pM) range 3. The detection process does not require complex instruments, making it suitable for portable devices. 4. Probes can be engineered for various pathogens, allowing customization. 5. Detection requires only microvolume samples, making it ideal for rare or difficult-to-obtain specimens.	1. Background signal interference can occur, especially in complex biological samples. 2. The performance of NanoBit sensors heavily depends on probe design.

Table references cited: [61,62,63,64,65].
